# Hamiltonian simulation for nonlinear partial differential equation by Schrödingerization

**DOI:** 10.1038/s41598-026-44920-8

**Published:** 2026-04-06

**Authors:** Shoya Sasaki, Katsuhiro Endo, Mayu Muramatsu

**Affiliations:** 1 Department of Science for Open and Environmental Systems, Keio University, 3-14-1 Hiyoshi, Yokohama, Kanagawa 223-8522 Japan; 2 Materials DX Research Center, National Institute of Advanced Industrial Science and Technology (AIST), Central 2, 1-1-1 Umezono, Tsukuba, Ibaraki 305-8568 Japan; 3 Department of Mechanical Engineering, Keio University, 3-14-1 Hiyoshi, Yokohama, Kanagawa 223-8522 Japan

**Keywords:** Quantum computing, Hamiltonian simulation, Schrödingerization, Warped phase transformation, Carleman linearization, Partial differential equations, Mathematics and computing, Physics

## Abstract

Hamiltonian simulation is a fundamental algorithm in quantum computing that has attracted considerable interest owing to its potential to efficiently solve the governing equations of large-scale classical systems. Exponential speedup through Hamiltonian simulation has been rigorously demonstrated in the case of coupled harmonic oscillators. The question arises as to whether Hamiltonian simulations in other physical systems also accelerate exponentially. Schrödingerization is a technique that transforms the governing equations of classical systems into the Schrödinger equation. However, since the Schrödinger equation is a linear equation, Hamiltonian simulation is often limited to linear equations. The research on Hamiltonian simulation methods for nonlinear governing equations remains relatively limited. In this study, we propose a Hamiltonian simulation method for nonlinear partial differential equations (PDEs). The proposed method is named Carleman linearization + Schrödingerization (CLS), which combines Carleman linearization (CL) and warped phase transformation (WPT). CL is first applied to transform a nonlinear PDE into a linear differential equation. This linearized equation is then mapped to the Schrödinger equation via WPT. The original nonlinear PDE can be solved efficiently by the Hamiltonian simulation of the resulting Schrödinger equation. By applying this method, we transform the original governing equation into the Schrödinger equation. Solving the transformed Schrödinger equation then enables the analysis of the original nonlinear equation. As a specific application, we apply this method to the nonlinear reaction–diffusion equation to demonstrate that Hamiltonian simulations are applicable to nonlinear PDEs.

## Introduction

Partial differential equations (PDEs) describe a wide range of physical phenomena, such as heat conduction, microstructure evolution in materials, and fluid dynamics. PDE-based analysis plays an important role in analyzing physical phenomena in the real world. One major challenge in PDE-based analysis is the difficulty in solving PDEs for extremely large-scale systems within practical time frames^1,2^. Quantum computing is a promising approach to overcoming this challenge. It has been increasingly attracting attention owing to its potential to accelerate the large-scale analysis of PDEs^3^. Quantum computing utilizes fundamental principles of quantum mechanics, such as superposition and entanglement, to perform calculations^4^. Compared with classical computing, quantum computing provides significant advantages in the analysis of large-scale PDEs^5,6^. Various methodologies have been investigated for simulating the time evolution of PDEs by quantum computing. These approaches can be broadly classified into two main categories.

The first category includes methods of solving difference equations obtained by discretizing time-dependent PDEs in the time and space directions using matrix operations. The first category involves the application of quantum algorithms originally developed for linear algebra problems^7,8^, such as the quantum linear systems algorithm (QLSA)^9–12^ including the Harrow–Hassidim–Lloyd algorithm^13–17^.

The second category includes Hamiltonian simulation methods^18–20^. The task of solving the time evolution of the solution to a Schrödinger equation for a time-independent Hamiltonian is called the Hamiltonian simulation problem^21^. The Hamiltonian simulation problem is rewritten in short as follows: give an initial state and Hamiltonian, and then find the time evolution of the solution. Since the Hamiltonian determines the time evolution of a system, the time evolution of various physical systems can be obtained by Hamiltonian simulation through the design of the Hamiltonian. Babbush et al.^22^ rigorously demonstrated an exponential quantum speedup by Hamiltonian simulation to a system of classical harmonic oscillators. Hamiltonian simulation is attracting attention as a method that has the potential to accelerate PDE-based analysis.

An analysis method based on nonlinear PDEs is also important because many real-world phenomena are nonlinear such as large deformation in materials, turbulence in fluid flow, chaotic systems and reaction–diffusion phenomena. The Hamiltonian simulation of nonlinear PDEs is difficult, and there has not been sufficient discussion or verification yet. There are two key challenges in performing Hamiltonian simulations of nonlinear PDEs.

Firstly, the target equation is a nonlinear PDE, whereas a Schrödinger equation is a linear PDE. This means that the target nonlinear PDEs must be transformed into a linear equation. Some algorithms for linearization that can be realized in quantum computing have been proposed. Joseph^23^ considered Koopman von Neumann (KvN) linearization based on the Koopman operator^24^ in quantum computing. KvN linearization is a general linearization method with a high degree of freedom in basis functions. Liu and coworkers^25,26^ applied Carleman linearization (CL)^25–32^ to linearize the nonlinear reaction–diffusion equation and employed QLSA to compute physical quantities such as energy. CL is a linearization method used when selecting polynomials as basis functions in KvN. Endo and Takahashi^33^ proposed an algorithm that mitigates the divergence of solutions caused by CL when implemented on a quantum computer. In this study, we focused on CL as a linearization method because it is a fundamental linearization method and has been extensively studied for its applications in quantum computing.

**Fig. 1:**
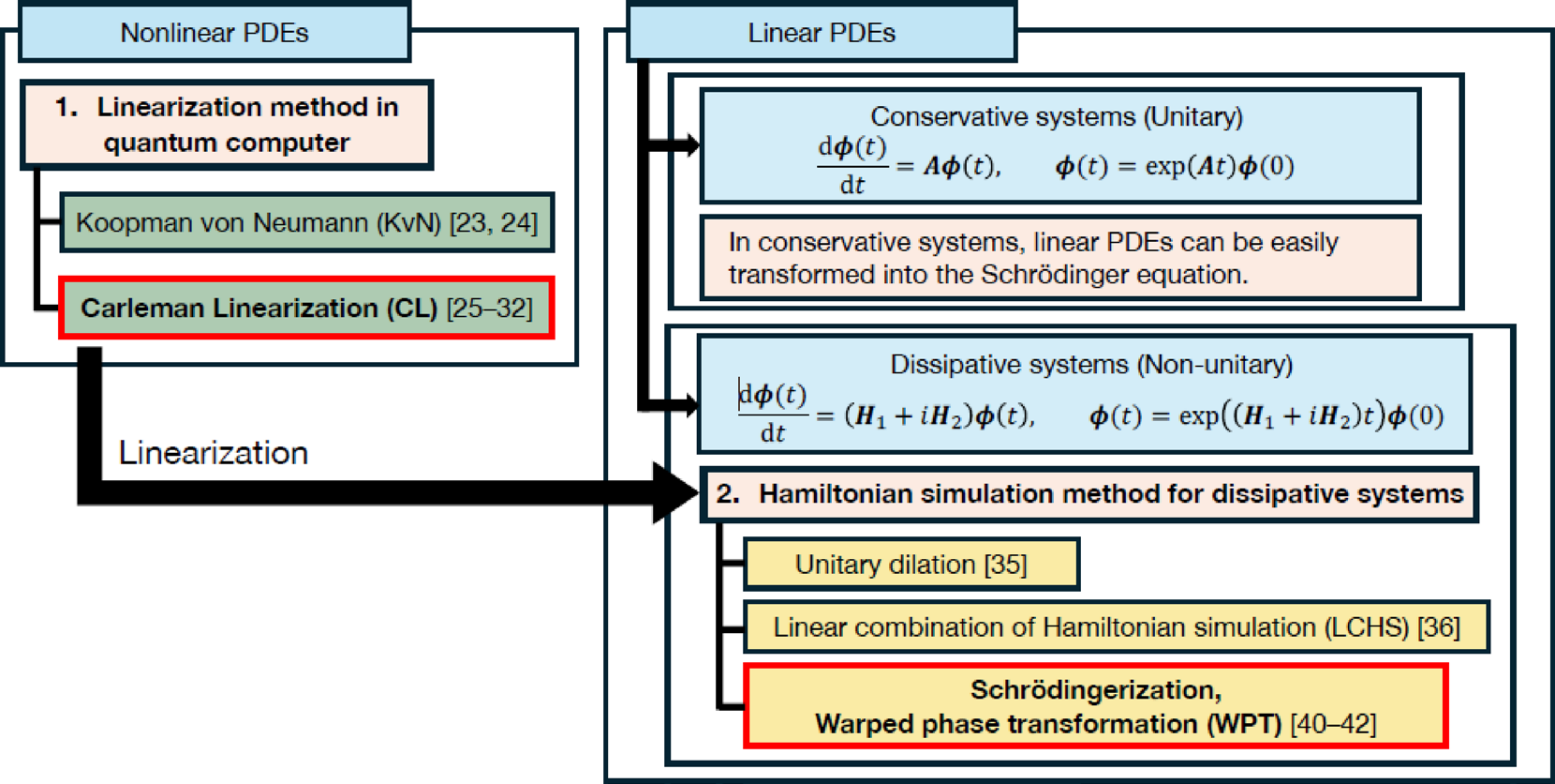
Two key challenges to performing Hamiltonian simulations of nonlinear PDEs and classification of previous methods for Hamiltonian simulation.

Secondly, a linearized equation is generally a dissipative system, whereas a Schrödinger equation is a conservative system. In this study, a conservative system is defined as the system with the time evolution operator represented by a unitarity operator. In contrast, a dissipative system is defined as the system with the time evolution operator represented by a non-unitarity operator. In conservative systems, linear PDEs can be easily transformed into a Schrödinger equation. Costa et al.^34^ proposed a quantum algorithm for simulating the wave equation under Dirichlet and Neumann boundary conditions, using Hamiltonian simulation as a subroutine. Sato et al.^1^ proposed a method of explicitly implementing quantum circuits for Hamiltonian simulation, applied it to linear advection and wave equations, and highlighted its potential for exponential speedup. However, these studies have been limited to conservative systems. Several methods have been proposed for handling dissipative systems on quantum computers. Gonzalez-Conde et al.^35^ converted the Black–Scholes equation in a dissipative system into the Schrödinger equation in a conservative system by unitary dilation. Unitary dilation introduces an additional ancilla qubit to the system, allowing the time evolution operator to be unitary. An et al.^36^ proposed the linear combination of Hamiltonian simulation (LCHS) as an approach to handling dissipative systems. LCHS can be viewed as a special case of linear combination of unitaries (LCU)^37–39^. Jin and coworkers^40–42^ proposed Schrödingerization, which is a method for mapping a general linear ordinary differential equation (ODE) including a dissipative system to a Schrödinger equation. The core of Schrödingerization is warped phase transformation (WPT), which converts a dissipative system into a conservative system by adding new auxiliary variables to the spatial dimensions of the system. In this study, we focused on WPT because unitary dilation and LCHS can only succeed in Hamiltonian simulation probabilistically, but WPT does not require probabilistic Hamiltonian simulation. However, it is unclear what advantages there are when postselection is included. In Fig. 1, two key challenges to performing Hamiltonian simulations of nonlinear PDEs are summarized and previous studies are classified.

In this study, we propose a method for the Hamiltonian simulation of nonlinear PDEs, named Carleman linearization + Schrödingerization (CLS). The proposed CLS framework integrates the following two components: first, CL is applied to transform the target nonlinear PDE into a system of linear ODEs. Then, the resulting linear system is converted into a Schrödinger equation using WPT. Finally, Hamiltonian simulation is applied to the transformed Schrödinger equation, allowing the time evolution of the original nonlinear PDE to be recovered.

In this study, we apply CLS to a nonlinear reaction–diffusion equation as a representative example of a nonlinear PDE. We evaluate the time evolution of the solution obtained by CLS, assess the associated errors, and verify the computational accuracy, thereby demonstrating the effectiveness of the CLS method.

## Theory

### Nonlinear reaction–diffusion equation

The nonlinear reaction–diffusion equation describes the time evolution of a system in which two processes, reaction and diffusion, proceed simultaneously. These processes include ecology, combustion, phase separation, and tissue formation phenomena. Letting *t* denote the time, $\boldsymbol{x}$ represent the spatial coordinates, and $\phi (t,\boldsymbol{x})$ the field variable, the reaction–diffusion equation is generally given by^43^

1 $$\begin{aligned} \frac{\partial {\phi }(t,\boldsymbol{x})}{\partial t}=D\nabla ^2{\phi }(t,\boldsymbol{x})+f({\phi }(t,\boldsymbol{x})), \end{aligned}$$

where $D\in \mathbb {R}_+$ is the diffusion coefficient and $f(\phi (t,\boldsymbol{x}))$ is an analytic function. For the purposes of this paper, we define a function as analytic if it can be expanded into a convergent Taylor series in a neighborhood of every point in its domain. This analyticity is required for the application of CL, which precludes the direct use of non-analytic functions such as piecewise functions. In Eq. (1), the term $D\nabla ^2\phi$ is the linear component, and $f(\phi (t,\boldsymbol{x}))$ corresponds to the nonlinear component. Therefore, the reaction–diffusion equation can be classified as a type of nonlinear PDE. In this study, we specifically focus on the KPP-Fisher equation, a fundamental reaction–diffusion equation model widely used in microstructure evolution in materials and biology. In this model $f(\phi (t,\boldsymbol{x}))$ is expressed as a quadratic function for $\phi$, given by

2 $$\begin{aligned} f(\phi (t,\boldsymbol{x}))=Q\phi (t,\boldsymbol{x})+R\phi (t,\boldsymbol{x})^2, \end{aligned}$$

where $Q\in \mathbb {R}$ is the coefficient of the first term and $R\in \mathbb {R}$ is the coefficient of the second term. Substituting Eq. (2) into Eq. (1), we obtain the following equation:

3 $$\begin{aligned} \frac{\partial \phi (t,\boldsymbol{x})}{\partial t}=D\nabla ^2\phi (t,\boldsymbol{x})+Q\phi (t,\boldsymbol{x})+R\phi (t,\boldsymbol{x})^2. \end{aligned}$$

We selected this exact quadratic form to demonstrate the validity of the CLS method on a system that is significant from a physical point of view. This avoids the severe divergence issues in CL that are often associated with high-order nonlinearities^26^, such as cubic terms or exponential functions that are approximated via polynomial expansion.

### CL

CL is a linearization technique that transforms a finite-dimensional nonlinear system into an infinite-dimensional linear system by extending the state variables into an infinite-dimensional space. In this section, we consider the application of CL to a general nonlinear differential equation. Nonlinear differential equations are generally expressed as

4 $$\begin{aligned} \frac{\textrm{d}\boldsymbol{x}}{\textrm{d}t}=\boldsymbol{f}(t,\boldsymbol{x}). \end{aligned}$$

Here, $\boldsymbol{x}=[x_1,\ldots ,x_n]^{\textrm{T}}\in \mathbb {R}^n$ is the state vector of the system and $\boldsymbol{f}(t,\boldsymbol{x}) \in \mathbb {R}^n$ is an analytic function of $\boldsymbol{x}$, defined as $\boldsymbol{f}:\mathbb {R}\times \mathbb {R}^n\rightarrow \mathbb {R}^n$. Approximating $\boldsymbol{f}(t,\boldsymbol{x})$ with a polynomial is given by

5 $$\begin{aligned} \boldsymbol{f}(t,\boldsymbol{x}(t))=\sum _{m=0}^\infty \boldsymbol{F}_m\boldsymbol{x}^{\otimes m}=\boldsymbol{F}_0+\boldsymbol{F}_1\boldsymbol{x}+\boldsymbol{F}_2\boldsymbol{x}^{\otimes 2}+\cdots , \end{aligned}$$

where $\otimes$ represents Kronecker’s product, $\boldsymbol{x}^{\otimes m}=\overbrace{\boldsymbol{x}\otimes \cdots \otimes \boldsymbol{x}}^{m~\text {times}}\in \mathbb {R}^{n^m}$ for any given non-negative integer *m*, and $\boldsymbol{F}_m\in \mathbb {R}^{n\times n^m}$ is a coefficient matrix of $\boldsymbol{x}^{\otimes m}$. For notational convenience, $\boldsymbol{x}^{\otimes 0}{:=}1$. The Kronecker product is an operation on two matrices of arbitrary size. The result of the operation is given as a matrix expanding the set of bases (i.e., $\otimes$: $(\mathbb {R}^{a}\times \mathbb {R}^{b}),(\mathbb {R}^{c}\times \mathbb {R}^{d})\rightarrow (\mathbb {R}^{ac}\times \mathbb {R}^{bd})$). In CL, we consider the time evolution of extended variables $\boldsymbol{y}_k{:=}\boldsymbol{x}^{\otimes k}$ for any positive integer *k*. According to Eqs. (4) and (5), and applying the chain rule, we obtain the time derivative of $\boldsymbol{y}_k$ as

6 $$\begin{aligned} \frac{\textrm{d}\boldsymbol{y}_k}{\textrm{d}t}=\frac{\textrm{d}\boldsymbol{x}^{\otimes k}}{\textrm{d}t}=\frac{\textrm{d}\boldsymbol{x}^{\otimes k}}{\textrm{d}\boldsymbol{x}}\frac{\textrm{d}\boldsymbol{x}}{\textrm{d}t}=\frac{\textrm{d}\boldsymbol{x}^{\otimes k}}{\textrm{d}\boldsymbol{x}}\sum _{m=0}^\infty \boldsymbol{F}_m\boldsymbol{x}^{\otimes m}. \end{aligned}$$

Applying the product rule of differentiation, we calculate $\textrm{d}\boldsymbol{x}^{\otimes k}/\textrm{d}\boldsymbol{x}$ as:

7 $$\begin{aligned} \frac{\textrm{d}\boldsymbol{x}^{\otimes k}}{\textrm{d}\boldsymbol{x}}=\sum _{v=0}^{k-1}\boldsymbol{x}^{\otimes v}\otimes \boldsymbol{I} \otimes \boldsymbol{x}^{\otimes k-1-v}. \end{aligned}$$

Substituting Eq. (7) into Eq. (6), we obtain

8 $$\begin{aligned} \frac{\textrm{d}\boldsymbol{y}_k}{\textrm{d}t}=\sum _{m=0}^\infty \left( \sum _{v=0}^{k-1}\boldsymbol{I}^{\otimes v}\otimes \boldsymbol{F}_m \otimes \boldsymbol{I}^{\otimes k-1-v}\right) \boldsymbol{x}^{\otimes m+k-1}. \end{aligned}$$

Here, we use the relationship $(\boldsymbol{A}\otimes \boldsymbol{B})(\boldsymbol{C}\otimes \boldsymbol{D})=\boldsymbol{AC}\otimes \boldsymbol{BD}$, which holds true when the matrices $\boldsymbol{A}, \boldsymbol{B}, \boldsymbol{C}$, and $\boldsymbol{D}$ are of a size such that the matrix products $\boldsymbol{AC}$ and $\boldsymbol{BD}$ can be defined. Here, by letting *l* be $m + k - 1$, we obtain the time evolution of $\boldsymbol{y}_k$ by calculating the following infinite-dimensional linear differential equation:

9 $$\begin{aligned} \frac{\textrm{d}\boldsymbol{y}_k}{\textrm{d}t}=\sum _{l=k}^\infty \boldsymbol{A}_{k,l}\boldsymbol{y}_l, \end{aligned}$$

where

10 $$\begin{aligned} \boldsymbol{A}_{k,l}=\sum _{v=0}^{k-1}\boldsymbol{I}^{\otimes v}\otimes \boldsymbol{F}_{l-k+1} \otimes \boldsymbol{I}^{\otimes k-1-v}. \end{aligned}$$

Eq. (9) is an infinite-dimensional linear differential equation. Since it is not possible to solve it in an infinite-dimensional space, we truncate Eq. (9) at the order of *K* and compute it as the following approximate finite-dimensional linear ODE:

11 $$\begin{aligned} \frac{\textrm{d}\boldsymbol{y}_k}{\textrm{d}t}=\sum _{l=k}^{K}\boldsymbol{A}_{k,l}\boldsymbol{y}_{l},~~~~1\le k\le K. \end{aligned}$$

Note that Eq. (11) is a dissipative system. To perform the Hamiltonian simulation of Eq. (11), a Hamiltonian simulation framework for dissipative systems is required.

### Schrödingerization using WPT

In this section, we introduce Schrödingerization framework which is a method for mapping a general linear ODE including a dissipative system to a Schrödinger equation. The core of Schrödingerization is WPT. In WPT, a dissipative system can be transformed into a conservative one by introducing an auxiliary variable independent of the spatial dimensions. We consider the application of WPT to a general linear ODE. A linear ODE is generally expressed as

12 $$\begin{aligned} \frac{\textrm{d}\boldsymbol{u}(t)}{\textrm{d}t}=\boldsymbol{A}\boldsymbol{u}(t), \end{aligned}$$

where $\boldsymbol{u}(t)\in \mathbb {C}^n$ is the state vector in the system and $\boldsymbol{A}\in \mathbb {C}^{n\times n}$ is a coefficient matrix. The WPT of dissipative systems into conservative systems is achieved by introducing an auxiliary variable independent of the spatial dimensions. First, since the coefficient matrix $\boldsymbol{A}$ is a square matrix, it is decomposed into its Hermitian part $\boldsymbol{H}_1$ and skew-Hermitian part $i\boldsymbol{H}_2$ as

13 $$\begin{aligned} \boldsymbol{A}=\boldsymbol{H}_1+i\boldsymbol{H}_2. \end{aligned}$$

The Hermitian part $\boldsymbol{H}_1$ and the skew-Hermitian part $i\boldsymbol{H}_2$ are defined as

14 $$\begin{aligned} \boldsymbol{H}_1=\frac{\boldsymbol{A}+\boldsymbol{A}^\dagger }{2},\quad i\boldsymbol{H}_2=\frac{\boldsymbol{A}-\boldsymbol{A}^\dagger }{2}. \end{aligned}$$

In WPT, we introduce an auxiliary variable, $p\ge 0$. WPT is formulated as

15 $$\begin{aligned} \boldsymbol{v}(t,p)=e^{-p}\boldsymbol{u}(t). \end{aligned}$$

Multiplying both sides of Eq. (12) by $e^{-p}$ yields

16 $$\begin{aligned} \frac{\textrm{d}}{\textrm{d}t}\left( e^{-p}\boldsymbol{u}(t)\right) =e^{-p}\boldsymbol{A}\boldsymbol{u}(t). \end{aligned}$$

Substituting Eq. (13) into Eq. (16) yields

17 $$\begin{aligned} \frac{\textrm{d}}{\textrm{d}t}\left( e^{-p}\boldsymbol{u}(t)\right) =e^{-p}\left( \boldsymbol{H}_1+i\boldsymbol{H}_2\right) \boldsymbol{u}(t). \end{aligned}$$

Eq. (17) can be transformed as

18 $$\begin{aligned} \frac{\textrm{d}}{\textrm{d} t}\left( e^{-p}\boldsymbol{u}(t)\right) =\left( -\boldsymbol{H}_1\frac{\partial }{\partial p}+i\boldsymbol{H}_2\right) e^{-p}\boldsymbol{u}(t). \end{aligned}$$

Substituting Eq. (15) into Eq. (18) yields

19 $$\begin{aligned} \frac{\textrm{d}\boldsymbol{v}(t,p)}{\textrm{d}t}=-\boldsymbol{H}_1\frac{\partial \boldsymbol{v}(t,p)}{\partial p}+i\boldsymbol{H}_2\boldsymbol{v}(t,p). \end{aligned}$$

The first term on the right-hand side of Eq. (19) captures the advection of $\boldsymbol{v}(t,p)$. Therefore, Eq. (19) should be discretized in the *p*-direction by the upwind difference method. The upwind difference method is a discretization technique in which the spatial differential term is approximated using the difference between a reference point and an upstream point. Furthermore, even if the initial value is extended to the region of $p<0$, the solution $\boldsymbol{v}(t,p)$ does not impact the region $p\ge 0$, because it flows from right to left in the *p*-direction. Therefore, we extend Eq. (19) to $p<0$ with the following initial condition:

20 $$\begin{aligned} \boldsymbol{v}(0,p)=e^{-|p|}\boldsymbol{u}(0). \end{aligned}$$

As a result, the ODE represented by Eq. (12) is transformed into the following system:

21 $$\begin{aligned} {\left\{ \begin{array}{ll} \frac{\textrm{d}\boldsymbol{v}(t,p)}{\textrm{d}t}=\boldsymbol{A}'\boldsymbol{v}(t,p),\\ \boldsymbol{v}(0,p)=e^{-|p|}\boldsymbol{u}(0), \end{array}\right. } \end{aligned}$$

where

22 $$\begin{aligned} \boldsymbol{A}'{:=}-\boldsymbol{H}_1\frac{\partial }{\partial p}+i\boldsymbol{H}_2. \end{aligned}$$

Note that the matrix $\boldsymbol{A}'$ is a skew-Hermitian matrix and the proof is shown in 5. Therefore, the time evolution of Eq. (21) is unitary and Eq. (21) is a Schrödinger equation. The unitarity of the time evolution of Eq. (21) implies that Eq. (12) is suitable for Hamiltonian simulation via WPT.

## Method

### CLS

**Fig. 2:**
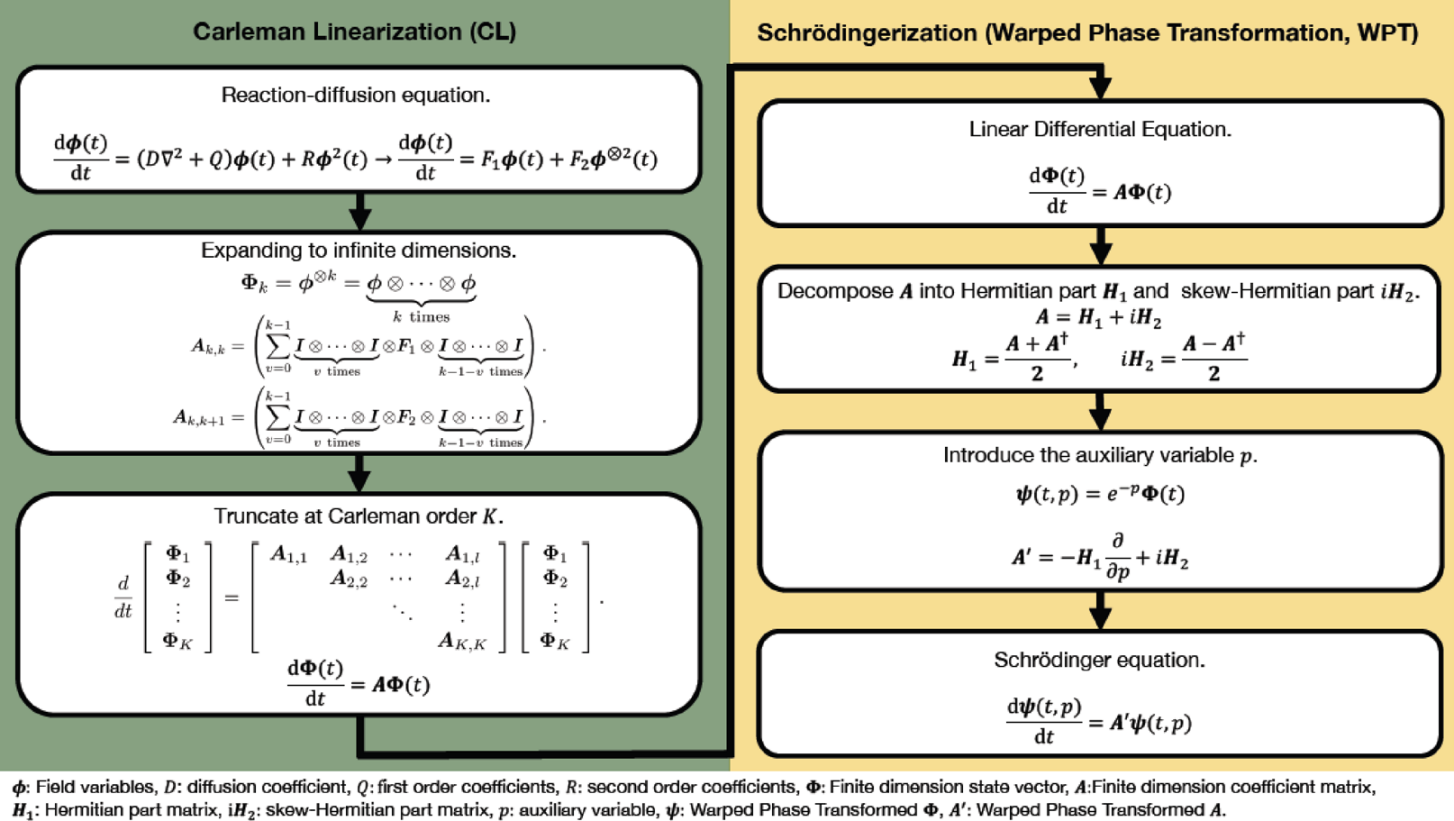
Flow of CLS.

Before detailing the mathematical procedures, it is noted that the fundamental objective of the applied transformations. The primary motivation of this study is to formulate a methodology for solving nonlinear PDEs on quantum computers. Because quantum mechanics natively processes only linear and unitary dynamics, the transformations applied in the CLS framework serve strictly as a necessary mathematical bridge, rather than a means to classically improve the original equation. In this study, we propose CLS as a Hamiltonian simulation method for nonlinear PDEs. Specifically, CL is used to transform a nonlinear PDE into a linear ODE, and WPT is used to transform a linearized ODE in a dissipative system into a Schrödinger equation. In this study, we apply CLS to nonlinear reaction–diffusion equations and examine its usefulness. First, we consider the discretization of the nonlinear reaction–diffusion equation shown in Eq. (3) in the *x*-direction. Let $\Omega _x{:=}(0, x_R)$ denote a one-dimensional spatial domain, and $x_R\in \mathbb {R_+}$ is the length of the spatial domain in the *x*-direction. We discretize the spatial domain $\Omega _x$ using $n_x$ grid points uniformly distributed with spacing $\Delta x=x_R/n_x$, where $n_x\in \mathbb {R}_+$ is the number of computational points in the *x*-direction. Then, the nonlinear diffusion-reaction equation is discretized as

23 $$\begin{aligned} \frac{\textrm{d}\boldsymbol{\phi }(t)}{\textrm{d}t}=(D\boldsymbol{\Delta }+Q)\boldsymbol{\phi }(t)+R\boldsymbol{\phi }(t)^2, \end{aligned}$$

where $\boldsymbol{\phi }(t)=\boldsymbol{\phi }=[\phi (t,x_0),\phi (t,x_1),\dots ,\phi (t,x_{n_x-1})]^{\textrm{T}}$ is the discretized field variable, $x_j$ for $j=0,1,\ldots ,n_x-1$ indicates the spatial coordinates of the *j*-th node of the *x*-direction, and $\boldsymbol{\Delta }$ is the Laplace operator discretized by the second-order central difference method. For notational convenience, we use $\phi _j$ given by $\phi _j{:=}\phi (t,x_j)$. Given the Dirichlet boundary conditions $\phi _{-1}=\phi _{n_x}=0$, the Laplace operator $\boldsymbol{\Delta }$ discretized by the second-order central difference method is as follows:

24 $$\begin{aligned} \boldsymbol{\Delta }=\frac{1}{(\Delta x)^2}\left[ \begin{array}{cccc} -2 & 1 & & \\ 1 & -2 & 1 & \\ & \ddots & \ddots & \ddots \\ & & 1 & -2 \end{array}\right] . \end{aligned}$$

Eq. (23) is also written as

25 $$\begin{aligned} \frac{\textrm{d}\boldsymbol{\phi }}{\textrm{d}t}=\boldsymbol{F}_1\boldsymbol{\phi }+\boldsymbol{F}_2\boldsymbol{\phi }^{\otimes 2}, \end{aligned}$$

where $\boldsymbol{F}_1=D\boldsymbol{\Delta }+Q$ and $\boldsymbol{F}_2$ is a linear mapping of $\boldsymbol{\phi }^{\otimes 2}$ to $R\boldsymbol{\phi }_j^2$ for $j=0,1,\ldots ,n_x-1$.

Subsequently, consider converting Eq. (25) into a linear ODE using CL. Let $\boldsymbol{\Phi }_k(t)= \boldsymbol{\phi }^{\otimes k}(t)$ and transform it into the following infinite-dimensional linear differential equation:

26 $$\begin{aligned} \frac{\textrm{d}\boldsymbol{\Phi }_k(t)}{\textrm{d}t}=\boldsymbol{A}_{k,k}\boldsymbol{\Phi }_k(t)+\boldsymbol{A}_{k,k+1}\boldsymbol{\Phi }_{k+1}(t). \end{aligned}$$

where $\boldsymbol{A}_{k,k}$ and $\boldsymbol{A}_{k,k-1}$ are respectively expressed as

27 $$\begin{aligned} \boldsymbol{A}_{k,k}=\sum _{v=0}^{k-1}\boldsymbol{I}^{\otimes v}\otimes \boldsymbol{F}_{1} \otimes \boldsymbol{I}^{\otimes k-1-v}, \end{aligned}$$

28 $$\begin{aligned}\boldsymbol{A}_{k,k+1}&=\sum _{v=0}^{k-1}\boldsymbol{I}^{\otimes v}\otimes \boldsymbol{F}_{2} \otimes \boldsymbol{I}^{\otimes k-1-v}. \end{aligned}$$

Since Eq. (26) is not solved in infinite dimensions, we truncate Eq. (26) at order *K* to obtain a finite-dimensional approximation. The approximate finite-dimensional linear differential equation truncated Eq. (26) at order *K* is

29 $$\begin{aligned} \frac{\textrm{d}\boldsymbol{\Phi }(t)}{\textrm{d}t}=\boldsymbol{A}{\boldsymbol{\Phi }(t)}, \end{aligned}$$

where $\boldsymbol{\Phi }$ and $\boldsymbol{A}$ are respectively defined as

30 $$\begin{aligned} \boldsymbol{\Phi }(t)=[\boldsymbol{\Phi }_1(t),\boldsymbol{\Phi }_2(t),\dots ,\boldsymbol{\Phi }_K(t)]^{\textrm{T}}, \end{aligned}$$

31 $$\begin{aligned} \boldsymbol{A}=\left[ \begin{array}{ccccc} {\boldsymbol{A}}_{1, 1} & {\boldsymbol{A}}_{1, 2} & & & \\ & {\boldsymbol{A}}_{2, 2}& {\boldsymbol{A}}_{2, 3}& & \\ & & \ddots & \ddots & \\ & & & {\boldsymbol{A}}_{K-1, K-1}& {\boldsymbol{A}}_{K-1, K}\\ & & & & {\boldsymbol{A}}_{K, K} \end{array}\right] . \end{aligned}$$

Matrix $\boldsymbol{A}$ is called the Carleman matrix^44^ and vector $\boldsymbol{\Phi }$ is called the Carleman state vector. The Carleman matrix is always a square matrix. Next, Eq. (29) is transformed into the Schrödinger equation using WPT. The Carleman matrix in Eq. (29) is decomposed into its Hermitian part $\boldsymbol{H}_1$ and skew-Hermitian part $i\boldsymbol{H}_2$. Then, the Carleman matrix that is not a skew-Hermitian matrix is

32 $$\begin{aligned} \boldsymbol{A}=\boldsymbol{H}_1+i\boldsymbol{H}_2. \end{aligned}$$

Introduce a new auxiliary variable $p\ge 0$ into the space variable *x* of the system. As described in Sec. 2.3, WPT is expressed as

33 $$\begin{aligned} \boldsymbol{\psi }(t,p)=e^{-p}\boldsymbol{\Phi }(t). \end{aligned}$$

According to Eq. (19), this variable $\boldsymbol{\psi }(t,p)$ satisfies the following equation:

34 $$\begin{aligned} \frac{\textrm{d}\boldsymbol{\psi }(t,p)}{\textrm{d}t}=\left( -\boldsymbol{H}_1\frac{\partial }{\partial p}+i\boldsymbol{H}_2\right) \boldsymbol{\psi }(t,p). \end{aligned}$$

Let $\Omega _p{:=}(p_L, p_R),~p_L<p_R$ denote a one-dimensional domain, and $p_L$ and $p_R$ be the endpoints of the domain in the *p*-direction. We discretize the spatial domain $\Omega _p$ using $n_p$ grid points uniformly distributed with spacing $\Delta p=(p_R-p_L)/n_p$, where $n_p\in \mathbb {R}_+$ is the number of computational points in the *p*-direction. $p_j$ for $j=0,1,\ldots ,n_p-1$ indicates the spatial coordinates of the *j*-th node of the *p*-direction. We define the following vector:

35 $$\begin{aligned} \boldsymbol{p}{:=}[e^{-p_0},e^{-p_1},\ldots ,e^{-p_{n_p-1}}]^{\textrm{T}}\in \mathbb {R}^{n_p}. \end{aligned}$$

Subsequently, we define $\boldsymbol{\psi }(t)$ as

36 $$\begin{aligned} \boldsymbol{\psi }(t){:=}\boldsymbol{p}\otimes \boldsymbol{\Phi }(t), \end{aligned}$$

where $\boldsymbol{\psi }(t)=[\boldsymbol{\psi }(t,p_0),\boldsymbol{\psi }(t,p_1),\ldots ,\boldsymbol{\psi }(t,p_{n_p-1})]^{\textrm{T}}$. For notational convenience, we use $\boldsymbol{\psi }_j(t)$ given by $\boldsymbol{\psi }_j(t){:=}\boldsymbol{\psi }(t,p_j)$. The variable $\boldsymbol{\psi }_j$ can be expressed as $\boldsymbol{\psi }_j(t)=e^{-p_j}\boldsymbol{\Phi }(t)$ for $j=0,1,\ldots ,n_p-1$. The first-order upwind difference method is used for the *p*-direction as the difference scheme in Eq. (34). The upwind difference method is a discretization technique in which the spatial differential term is approximated by the difference between a reference point and an upstream point. Since Eq. (34) advects in the *p*-negative direction, applying the upwind difference method yields

37 $$\begin{aligned} \frac{\textrm{d}\boldsymbol{\psi }_j(t)}{\textrm{d} t}=-\boldsymbol{H}_1\frac{\boldsymbol{\psi }_{j+1}(t)-\boldsymbol{\psi }_{j}(t)}{\Delta p}+i\boldsymbol{H}_2\boldsymbol{\psi }_j(t). \end{aligned}$$

In this study, the time evolution of the solution of the nonlinear reaction–diffusion equation by CLS is obtained by time evolving Eq. (37).

We consider another representation of Eq. (37). Applying a left tensor product with $\boldsymbol{p}$ to both sides of Eq. (29) yields

38 $$\begin{aligned} \frac{\textrm{d}}{\textrm{d}t}(\boldsymbol{p}\otimes \boldsymbol{\Phi }(t))=\boldsymbol{p}\otimes \boldsymbol{H}_1\boldsymbol{\Phi }(t)+\boldsymbol{p}\otimes i\boldsymbol{H}_2\boldsymbol{\Phi }(t). \end{aligned}$$

Eq. (38) can be transformed into

39 $$\begin{aligned} \frac{\textrm{d}}{\textrm{d}t}(\boldsymbol{p}\otimes \boldsymbol{\Phi }(t))=-\boldsymbol{\nabla }_p\boldsymbol{p}\otimes \boldsymbol{H}_1\boldsymbol{\Phi }(t)+\boldsymbol{p}\otimes i\boldsymbol{H}_2\boldsymbol{\Phi }(t), \end{aligned}$$

where $\boldsymbol{\nabla }_p$ is the gradient operator in the *p*-direction discretized by the first-order upwind difference method. Given the periodic boundary condition, considering the advection from right to left in the *p* domain, the upwind difference method is defined as

40 $$\begin{aligned} \boldsymbol{\nabla }_p=\frac{1}{\Delta p} \begin{bmatrix} -1 & 1 & & & \\ & -1 & 1 & & \\ & & \ddots & \ddots & \\ & & & -1 & 1\\ 1 & & & & -1 \end{bmatrix}. \end{aligned}$$

Eq. (39) can be transformed into

41 $$\begin{aligned} \frac{\textrm{d}}{\textrm{d}t}(\boldsymbol{p}\otimes \boldsymbol{\Phi }(t))=(-\boldsymbol{\nabla }_p\otimes \boldsymbol{H}_1+\boldsymbol{I}\otimes i\boldsymbol{H}_2)(\boldsymbol{p}\otimes \boldsymbol{\Phi }(t)). \end{aligned}$$

Substituting Eq. (36) into Eq. (41) yields

42 $$\begin{aligned} \frac{\textrm{d}\boldsymbol{\psi }}{\textrm{d}t}=\tilde{\boldsymbol{H}}\boldsymbol{\psi }, \end{aligned}$$

where

43 $$\begin{aligned} \tilde{\boldsymbol{H}}{:=}-\boldsymbol{\nabla }_p\otimes \boldsymbol{H}_1+\boldsymbol{I}\otimes i\boldsymbol{H}_2. \end{aligned}$$

Eqs. (42) and (43) are other representations of Eq. (37). When we consider the implementation of CLS on quantum circuits, the representations of Eqs. (42) and (43) are more suitable than that of Eq. (37). We extend Eqs. (37) and (42) to $p<0$ ($p_L<0, p_R>0$) with the following initial data:

44 $$\begin{aligned} \boldsymbol{\psi }(0)=\boldsymbol{P}\otimes \boldsymbol{\Phi }(0), \end{aligned}$$

where

45 $$\begin{aligned} \boldsymbol{P}=[e^{-|p_0|},e^{-|p_1|},\ldots ,e^{-|p_{n_p-1}|}]^{\textrm{T}}\in \mathbb {R}^{n_p}. \end{aligned}$$

It is important to note that errors in preserving the macroscopic properties of the original equation are not fundamental deteriorations caused by the transformations themselves. Instead, such errors originate strictly from numerical approximations, specifically the finite truncation of the CL and the spatial discretization and advection effects in the WPT. By strictly managing the computational conditions, such as increasing the truncation order and refining the grid resolution, these numerical deviations can be systematically suppressed.

Furthermore, although this study focuses on the application of CLS to a nonlinear reaction-diffusion equation, the proposed framework can be applied to a wider range of PDEs, including hyperbolic and elliptic PDEs. To apply CLS to such equations, the original PDEs must first be reformulated as a system of first-order ordinary differential equations using spatial discretization and introducing additional state variables for higher-order time derivatives. If the nonlinear terms in this reduced first-order system are analytic, CLS can be applied directly. However, it should be noted that this method cannot be applied directly to stochastic differential equations because the stochastic terms are inherently non-analytic, which violates the fundamental assumption of CL.

### Classical numerical methods for CLS

The discretization in the *x*- and *p*-directions discussed in Section 3.1, which was originally in the context of classical computation, can also be applied when implementing the method on a quantum computer. Since Hamiltonian simulation operates analogously rather than digitally, it does not update the state step-by-step in time during simulations; instead, it can directly generate the dynamics corresponding to the desired time evolution in an analog manner. In other words, when considering the implementation of CLS on a quantum computer, the time step size in classical simulation can effectively be set to zero. Note that, while this eliminates the time step error inherent in classical simulation, it does not render the quantum simulation error-free. Other types of error may arise depending on the quantum algorithm employed, such as time discretization errors in Suzuki–Trotter decompositions or approximation errors in the construction of time-evolution operators via quantum singular value transformation^45^.

However, in this study, the primary objective is to investigate the utility and characteristics of CLS through classical simulations. Therefore, for classical simulations, we consider discretization in the time direction. Specifically, we examine the time evolution from the initial time $t = 0$ up to the integration time $t = T$. Letting $n_t$ denote the number of time steps, we can express the time step size $\Delta t$ as $\Delta t = T / n_t$. By applying the first-order forward difference scheme to the time derivative term in Eq. (37), we obtain the following expression:

46 $$\begin{aligned} \frac{\boldsymbol{\psi }_j{(n+1)}-\boldsymbol{\psi }_j{(n)}}{\Delta t}=-\boldsymbol{H}_1\frac{\boldsymbol{\psi }_{j+1}{(n)}-\boldsymbol{\psi }_{j}{(n)}}{\Delta p}+i\boldsymbol{H}_2\boldsymbol{\psi }_j{(n)}. \end{aligned}$$

Here, $n=0,1,\ldots ,n_t-1$ is the number of time steps and $\boldsymbol{\psi }_j(n)$ represents $\boldsymbol{\psi }_j(t)$ at $t=n\Delta t$. Rearranging with respect to $\boldsymbol{\psi }_j(n)$, we obtain the following equation:

47 $$\begin{aligned} \boldsymbol{\psi }_j{(n+1)}=-\boldsymbol{H}_1\frac{\Delta t}{\Delta p}\boldsymbol{\psi }_{j+1}{(n)}+\left( 1+\boldsymbol{H}_1\frac{\Delta t}{\Delta p}+i\boldsymbol{H}_2\Delta t\right) \boldsymbol{\psi }_j{(n)}. \end{aligned}$$

By introducing $\boldsymbol{B}_1$ and $\boldsymbol{B}_2$, we can rewrite the equation as

48 $$\begin{aligned} \boldsymbol{\psi }_j{(n+1)}=\boldsymbol{B}_1\boldsymbol{\psi }_{j+1}{(n)}+\boldsymbol{B}_2\boldsymbol{\psi }_j{(n)}, \end{aligned}$$

where

49 $$\begin{aligned} \boldsymbol{B}_1{:=}-\boldsymbol{H}_1\frac{\Delta t}{\Delta p},\quad \boldsymbol{B}_2{:=}1+\boldsymbol{H}_1\frac{\Delta t}{\Delta p}+i\boldsymbol{H}_2\Delta t. \end{aligned}$$

Given the boundary condition $\boldsymbol{\psi }_0=\boldsymbol{\psi }_{n_p}$, the final iterative system is as follows:

50 $$\begin{aligned} \boldsymbol{\psi }(n+1)=\boldsymbol{B}\boldsymbol{\psi }(n),\quad n=0,1,\ldots ,n_t-1, \end{aligned}$$

where

51 $$\begin{aligned} \boldsymbol{B}= \begin{bmatrix} \boldsymbol{B}_2 & \boldsymbol{B}_1 & & & \\ & \boldsymbol{B}_2 & \boldsymbol{B}_1 & & \\ & & \ddots & \ddots & \\ & & & \boldsymbol{B}_2 & \boldsymbol{B}_1\\ \boldsymbol{B}_1& & & & \boldsymbol{B}_2 \end{bmatrix}. \end{aligned}$$

By iteratively computing Eq. (50), we can obtain the solution vector of $\boldsymbol{\psi }$ at the desired time.

**Table 1:** Computational conditions for FDM, CL, and CLS.

Computation domain of *x*	$x\in (0\mathrm {\,m},1\mathrm {\,m})$
Computation domain of *p*	$p\in [p_L,p_R]=[-20\mathrm {\,m},20\mathrm {\,m}]$
Number of calculation points for *x*	$n_x=36$
Number of calculation points for *p*	$n_p=256$
Discretization method for *x*	Second-order central difference method
Discretization method for *p*	First-order upwind difference method
Time step size $\varDelta t$	$\varDelta t=1.0\times 10^{-6}\mathrm {\,s}$
Number of time steps $n_t$	$n_t=0.4 \times 10^6$
Initial distribution $\phi (0,x)$	$\phi (0,x)=0.5-0.5\cos (2\pi x)$
Boundary condition for *x*	Dirichlet condition ($\phi _{-1}=\phi _{n_x}=0$)
Boundary condition for *p*	Periodic condition ($\boldsymbol{\psi }_0=\boldsymbol{\psi }_{n_p}$)
CL truncation order *K*	3
Variables in equation *P*, *Q*, *R*	$P=1,~Q=1,~R=-1$

## Results and discussion

The nonlinear reaction–diffusion equation shown in Eq. (1) is analyzed by the proposed method CLS shown in Fig. 2. In this study, to investigate the usefulness of CLS, a discretized and time-evolved version of the finite differential method (FDM) using the central difference method and a time-evolved version of the linearized equation using CL were prepared and compared. The validity of the proposed method was evaluated by determining the accuracy of calculation by CLS. The computational conditions in this study are shown in Table 1.

To clarify the objective of these experiments, it is emphasized that the CLS method is not intended to compete with traditional numerical methods, such as the finite difference method. Rather, the classical simulations of the CLS framework presented here provide a rigorous theoretical proof of concept for quantum simulation. Since quantum computers can only natively execute linear and unitary operations, it is critical to evaluate the algorithmic errors that arise from transforming a nonlinear PDE into a quantum-solvable format. Validating these properties on classical hardware strictly isolates them from the physical noise inherent in current quantum devices.

**Fig. 3:**
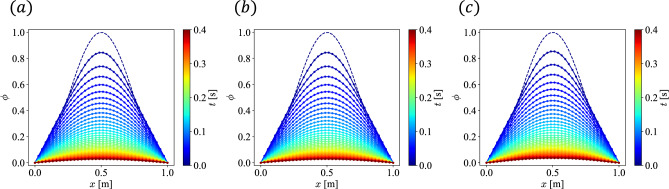
Time evolution of the solution of the nonlinear reaction–diffusion equation: (**a**) solution of FDM, (**b**) solution of CL, and (**c**) solution of CLS. Dashed lines represent the initial condition.

**Fig. 4:**
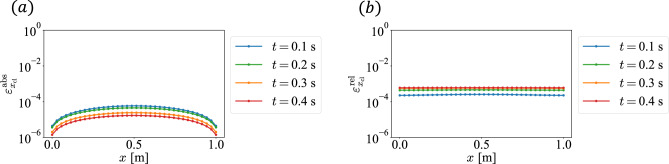
Error between CL and FDM based on the dynamics of the nonlinear reaction–diffusion equation: (**a**) absolute error $\varepsilon _{x_{\text {cl}}}^{\text {abs}}$ and (**b**) relative error $\varepsilon _{x_{\text {cl}}}^{\text {rel}}$.

**Fig. 5:**
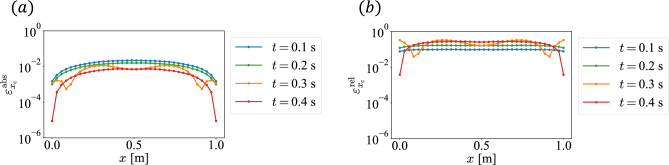
Error between CL and CLS: (**a**) absolute error $\varepsilon _{x_{\text {c}}}^{\text {abs}}$ and (**b**) relative error $\varepsilon _{x_{\text {c}}}^{\text {rel}}$.

**Fig. 6:**
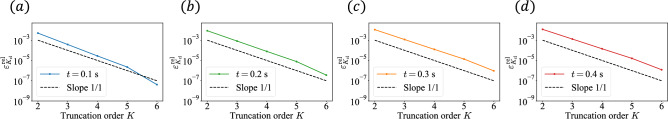
Relationship between the truncation order of CL and the relative error for the nonlinear reaction–diffusion equation solved using CL: (**a**) relative error at $t=0.1\mathrm {\,s}$, (**b**) relative error at $t=0.2\mathrm {\,s}$, (**c**) relative error at $t=0.3\mathrm {\,s}$, and (**d**) relative error at $t=0.4\mathrm {\,s}$.

**Fig. 7:**
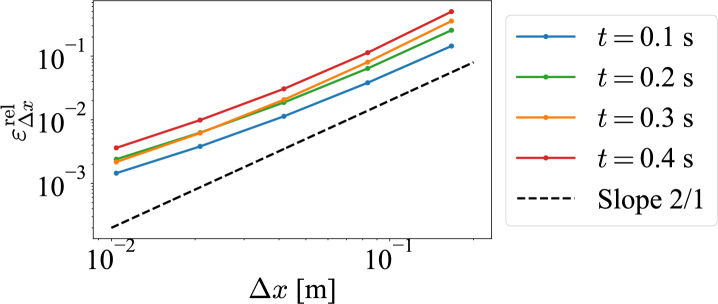
Computational accuracy of CLS with respect to $\varDelta x$.

**Fig. 8:**
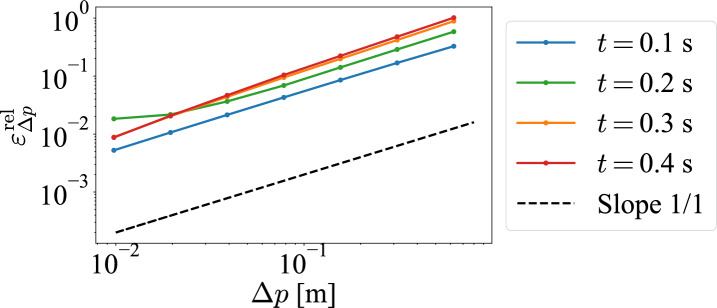
Computational accuracy of CLS with respect to $\Delta p$.

**Fig. 9:**
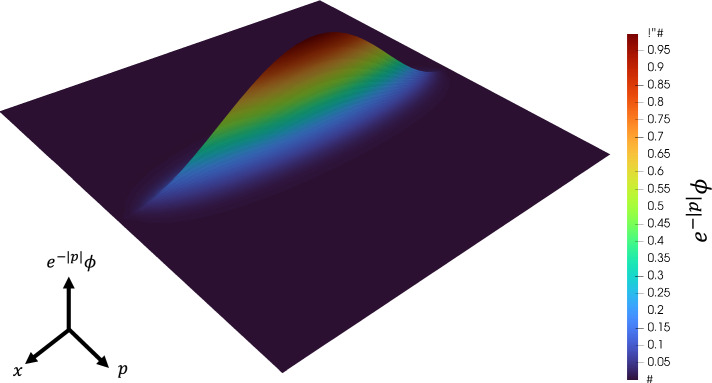
3D visualization of initial distribution $\boldsymbol{\psi }(0)$.

**Fig. 10:**
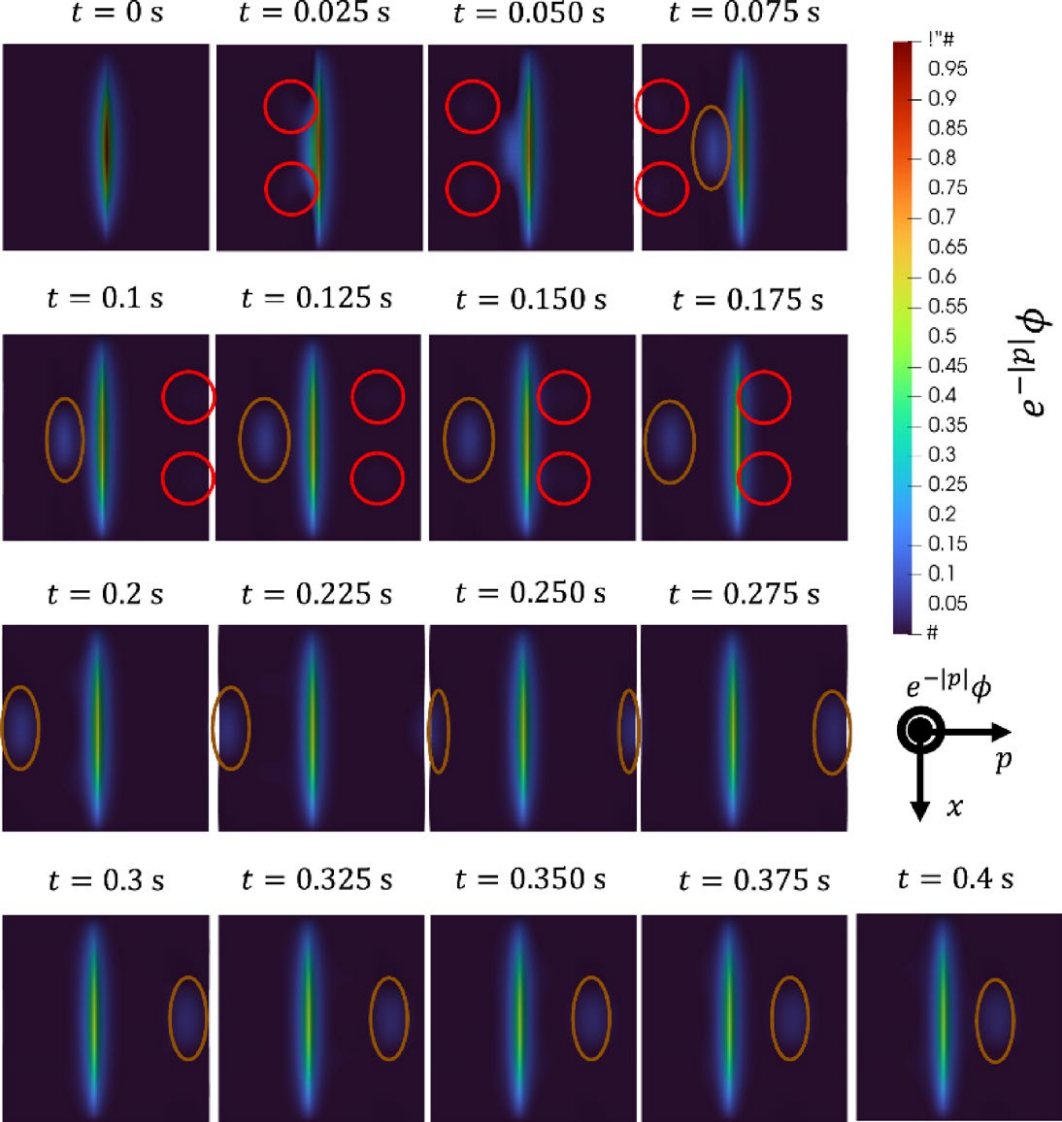
Time evolution of the solution obtained by CLS on the *x*-*p* plane.

### Time evolution of the solution by CLS

The time evolution of the solutions of Eq. (3) by FDM, CL and CLS are shown in Fig. 3a–c, respectively. It can be seen that the time evolution of the solution by the proposed method CLS qualitatively coincides with those of the solutions by FDM and CL in Fig. 3a and b.

### Accuracy of CLS calculations

In this section, we discuss the accuracy of CLS calculations. The error of CL with FDM as the true value is shown in Fig. 4, and that of CLS with CL as the true value is shown in Fig. 5. From Figs. 4 and 5, we see that the error due to CLS is larger than that due to CL. Therefore, it can be assumed that the error due to CLS is dominated by that introduced by the WPT process.

Next, we investigated the accuracy of the calculation for the truncated order *K* of CL. The relative error of CL when FDM is taken as the true value for the truncated order *K* of CL is shown in Fig. 6. This relative error specifically represents the trucation error of CL up to truncation order *K*. From Fig. 6, we see that the relative error is parallel to the line with slope 1/1 on both logarithmic plots. Therefore, we can consider that CL is first-order-accurate for the truncated order *K*.

Next, the accuracy of the calculation is investigated for the *x*-direction. The relative error of CLS when FDM is taken as the true value for the spatial step size in the *x*-direction, $\Delta x$, is shown in Fig. 7. From Fig. 7, we see that the relative error is parallel to the straight line with slope of 2/1 on both logarithmic plots. CLS shows second-order accuracy for the *x* spatial step size $\Delta x$. This result implies that it is based on the discretization using a second-order central difference method for the *x*-direction.

Next, we examine the accuracy of the calculation for the *p*-direction. The relative error of CLS when CL is the true value for CLS with respect to the spatial step size in the *p*-direction $\Delta p$ is shown in Fig. 8. From Fig. 8, we see that the relative error is parallel to the line with slope 1 on both logarithmic plots. Therefore, we can consider that CLS is first-order-accurate for the *p* spatial step size $\Delta p$. This result implies that it is based on the discretization using the first-order upwind difference method for the *p*-direction.

Furthermore, comparing the relative errors in Figs. 6, 7, and 8 reveals a clear pattern regarding the error sources. The error introduced by the CL truncation, which is on the order of $10^{-5}$ to $10^{-3}$ for $K=3$, is significantly smaller than the discretization errors in the *x* and *p* directions, which are on the order of $10^{-3}$ to $10^{-1}$. This confirms that increasing the truncation order *K* systematically improves the accuracy of the CL portion. However, the overall accuracy of the CLS method is currently dominated by the spatial discretizations, particularly the advection in the WPT process.

We consider the error caused by advection in WPT. In WPT, the solution advects in the direction of $p<0$. Fig. 9 shows a 3D plot of the initial distribution. Figure 10 shows the time evolution of the solution obtained by CLS on the *x*-*p* plane. The red circle surrounds the wave that first affects the calculation accuracy. The brown circle surrounds the wave that next affects the calculation accuracy. Note that periodic boundary conditions are imposed in the *p*-direction. The red and brown circles have different propagation speeds. Although the computational domain is extended to the $p < 0$ region under the assumption that it does not affect the solution in the $p \ge 0$ region, it is considered that this affects the computational accuracy because it affect the $p \ge 0$ region. For long-term simulations, the errors caused by this advection in WPT can be a problem.

## Conclusion

In this study, the proposed CLS method was applied to the nonlinear reaction–diffusion equation, and the time evolution of the solution by CLS and its computational accuracy were investigated. The time evolution of the solution by CLS was almost the same as that by the conventional method. The computational accuracy of CLS was found to be first-order accuracy for the truncated order of CL, second-order accuracy for the spatial variable *x*-direction, and first-order accuracy for the auxiliary variable *p*-direction. The computational accuracy in the *x*- and *p*-directions was considered to be the result of discretization by the second-order accuracy central difference and first-order accuracy upwind difference methods, respectively. This indicated that the computations performed using CLS are consistent with the theoretical predictions. The proposed CLS method extended the framework of time evolution simulation in quantum computing and newly shows that Hamiltonian simulation can be applied to nonlinear PDEs.

This study focused on demonstrating the fundamental validity of the CLS framework using a nonlinear reaction-diffusion equation. However, the ultimate advantage of this quantum simulation approach lies in its application to extremely large-scale physical systems governed by analytic nonlinearities. Babbush et al.^22^ rigorously demonstrated that Hamiltonian simulation can provide an exponential quantum speedup, reducing the computational cost from $\textrm{poly}(N)$ in classical methods to $\textrm{polylog}(N)$ for simulating *N* coupled classical harmonic oscillators. Here, $\textrm{poly}(N)$ and $\textrm{polylog}(N)$ denote $O(N^m)$ and $O(\log ^m(N))$, respectively, for some constant *m*. We expect that similar exponential advantages to be achievable for nonlinear systems using the CLS framework. The CLS approach would benefit physical systems requiring massive degrees of freedom, such as large-scale chemical reaction networks, complex combustion processes, and phase separation phenomena. Using the CLS method on these high-dimensional, computationally demanding systems is a promising way to achieve practical quantum advantage.

While the proposed CLS method demonstrates the applicability of Hamiltonian simulation to nonlinear PDEs, certain challenges remain. First, because the CL relies on polynomial expansion, the CLS method is currently limited to analytic nonlinearities; systems with stochastic terms or discontinuities cannot be directly processed. Second, the stability of the method is highly sensitive to the chosen boundary conditions. Although Dirichlet boundary conditions yield stable results, our investigations indicate that applying periodic boundary conditions in the spatial direction causes the solutions of both CL and CLS to diverge. Addressing these limitations to accommodate broader classes of nonlinearities and boundary conditions will be the focus of future work.

## Data Availability

The datasets generated and/or analysed during the current study are available in the $\hbox {CLS}\_\hbox {dataset}$ repository, https://github.com/mmc-research-group/CLS_dataset.

## Appendix: Skew-Hermitianity of $\boldsymbol{A}'$

The operator $\boldsymbol{A}'$ obtained from WPT is

52 $$\begin{aligned} \boldsymbol{A}'=-\boldsymbol{H}_1\frac{\partial }{\partial p}+i\boldsymbol{H}_2, \end{aligned}$$

where $\boldsymbol{H}_1$ is the Hermitian operator and $\boldsymbol{H}_2$ is the skew-Hermitian operator. We define $\mathbb {R}_p=\{p\in \mathbb {R}\}$ and consider the following Hilbert space $\mathscr {H}$:

$$\begin{aligned} \mathscr {H}&=L^2(\mathbb {R}_p)\nonumber =\left\{ f:\mathbb {R}\rightarrow \mathbb {C}~\left| ~\int _{-\infty }^{\infty }|f(p)|^2dp<\infty \right. \right\} . \end{aligned}$$

At this time, the inner product is defined as

53 $$\begin{aligned} \langle \varphi ,\psi \rangle =\int _{-\infty }^{\infty }\varphi ^*(p)\psi (p)dp,\quad \varphi ,\psi \in \mathscr {H}, \end{aligned}$$

where $\varphi ^*(p)$ is the complex conjugate of $\varphi (p)$. When an operator $\boldsymbol{G}$ satisfies the following equation, $\boldsymbol{G}$ is called a skew-Hermitian operator:

54 $$\begin{aligned} \langle \varphi ,\boldsymbol{G}\psi \rangle =-\langle \boldsymbol{G}\varphi ,\psi \rangle ,\quad \varphi ,\psi \in \mathscr {H}. \end{aligned}$$

Consider the following inner product to confirm the skew-Hermitian property of the operator $\partial /\partial p$:

55 $$\begin{aligned} \left\langle \varphi ,\frac{\partial \psi }{\partial p}\right\rangle&=\int _{-\infty }^{\infty }\varphi ^*(p)\frac{\partial \psi (p)}{\partial p}dp,\nonumber \\&=\left[ \varphi ^*(p)\psi (p)\right] _{-\infty }^{\infty }-\int _{-\infty }^{\infty }\frac{\partial \varphi ^*(p)}{\partial p}\psi (p)dp,\nonumber \\&=-\int _{-\infty }^{\infty }\left( \frac{\partial \varphi (p)}{\partial p}\right) ^*\psi (p)dp,\nonumber \\&=-\left\langle \frac{\partial \varphi }{\partial p},\psi \right\rangle . \end{aligned}$$

Therefore, the operator $\partial /\partial p$ is a skew-Hermitian operator. The complex transpose of operator $\boldsymbol{A}'$ is as follows:

56 $$\begin{aligned} \boldsymbol{A}'^\dagger&=\left( -\boldsymbol{H}_1\frac{\partial }{\partial p}+i\boldsymbol{H}_2\right) ^\dagger ,\nonumber \\&=\left( -\boldsymbol{H}_1\frac{\partial }{\partial p}\right) ^\dagger +(i\boldsymbol{H}_2)^\dagger ,\nonumber \\&=-\left( \frac{\partial }{\partial p}\right) ^\dagger \boldsymbol{H}_1^\dagger -i\boldsymbol{H}_2,\nonumber \\&=\frac{\partial }{\partial p}\boldsymbol{H}_1-i\boldsymbol{H}_2,\nonumber \\&=-\left( \boldsymbol{H}_1\frac{\partial }{\partial p}+i\boldsymbol{H}_2\right) ,\nonumber \\&=-\boldsymbol{A}'. \end{aligned}$$

Therefore, $\boldsymbol{A}'$ is a skew-Hermitian operator.
